# Immune profiling in trigeminal neuralgia reveals altered cytokine and chemokine signatures in cerebrospinal fluid and serum

**DOI:** 10.3389/fphar.2026.1796130

**Published:** 2026-05-08

**Authors:** Hong Liu, Zi-Wei Liu, Jiang-Tao Zhang, Xue-Long Wang, Li-Hua Chen, Xiao-Hong Jin, Tong Liu, Fu-Hai Ji

**Affiliations:** 1 Department of Anesthesiology and Pain Management, The First Affiliated Hospital of Soochow University, Suzhou, China; 2 Institute of Pain Medicine and Special Environmental Medicine, Nantong University, Nantong, China; 3 Department of Thoracic Surgery, Capital Medical University Electric Power Teaching Hospital Beijing, Beijing, China; 4 Department of Nutrition and Food Hygiene, School of Public Health, Nantong University, Nantong, China; 5 College of Life Sciences, Yanan University, Yanan, China

**Keywords:** cerebrospinal fluid, chemokine, cytokine, neuroinflammation, trigeminal neuralgia

## Abstract

**Introduction:**

Trigeminal neuralgia (TN) is a debilitating neuropathic pain disorder traditionally attributed to neurovascular compression; however, this mechanism does not fully explain the marked clinical heterogeneity or persistent pain observed in a subset of patients. Increasing evidence suggests that central neuroinflammatory processes contribute to neuropathic pain, but immune profiles in the cerebrospinal fluid (CSF) and serum of patients with TN remain poorly characterized.

**Methods:**

We conducted a single-center cross-sectional study including 14 patients with TN and 19 non-pain controls. CSF samples were obtained from 14 TN patients and 10 controls, while serum samples were available from 10 TN patients and 9 controls. Concentrations of a broad panel of cytokines, chemokines, and growth factors were measured using multiplex immunoassays. Group comparisons were performed using non-parametric statistical tests, with false discovery rate correction for multiple comparisons.

**Results:**

Patients with TN reported severe pain (median numerical rating scale score: 8), whereas controls reported no pain. Demographic characteristics, including age, sex, and body mass index, were comparable between groups. Compared with controls, CSF from TN patients showed significantly elevated levels of multiple pro-inflammatory cytokines and chemokines, including TNF-α TNF-β, IL-1β, IL-9, IL-16, IL-17, IL-18, IFN-γ, CCL5, CXCL12, and macrophage migration inhibitory factor (MIF) (all false discovery rate-adjusted p < 0.05). In contrast, vascular endothelial growth factor (VEGF) concentrations were significantly reduced in the CSF of TN patients. No corresponding inflammatory alterations were observed in serum samples.

**Discussion:**

TN is associated with a distinct CSF immune signature characterized by elevated pro-inflammatory mediators and altered growth factor profiles. These findings support the involvement of central neuroimmune mechanisms in the pathophysiology of TN and highlight the potential value of CSF biomarkers for improving mechanistic understanding and identifying novel therapeutic targets.

## 1 Introduction

Trigeminal neuralgia (TN) is a severe neuropathic pain disorder characterized by recurrent, brief, electric shock–like facial pain attacks affecting one or more branches of the trigeminal nerve. Despite its relatively low prevalence, TN imposes a profound burden on patients’ quality of life and is frequently associated with substantial disability, psychological distress, and impaired social functioning (Truini, 2015; Maarbjerg et al., 2017; Bendtsen et al., 2019; Cruccu et al., 2020).

Neurovascular compression of the trigeminal nerve root entry zone has long been regarded as the principal pathological mechanism underlying classical TN (Love and Coakham, 2001; Baron et al., 2010). Chronic pulsatile vascular contact is thought to induce focal demyelination, ectopic impulse generation, and ephaptic transmission, ultimately giving rise to paroxysmal pain attacks (Devor et al., 2002). This model is supported by neuroimaging findings and by the clinical efficacy of microvascular decompression in selected patients (Bendtsen et al., 2019). However, neurovascular compression alone does not fully account for the clinical heterogeneity of TN (Guan et al., 2025; Wang et al., 2025). Not all individuals with radiological evidence of vascular contact develop TN, and a subset of patients continue to experience persistent or recurrent pain despite technically successful decompression surgery (Cruccu et al., 2016; Maarbjerg et al., 2017). These observations suggest that alternative mechanisms beyond structural compression may contribute to pain generation and maintenance in TN.

Accumulating evidence indicates that neuroinflammatory processes play a critical role in the pathophysiology of neuropathic pain (Huang et al., 2020; Park et al., 2023; Cai et al., 2025). Activation of glial cells and the release of pro-inflammatory cytokines and chemokines within the central nervous system can enhance neuronal excitability, facilitate maladaptive synaptic plasticity, and promote central sensitization (Grace et al., 2014; Ji et al., 2018). Cytokines such as tumor necrosis factor (TNF), interleukins (e.g., IL-1β, IL-6, IL-17), and interferon-γ have been implicated in sustaining pain signaling across diverse neuropathic pain conditions (Raoof et al., 2018; Sommer et al., 2018).

In the context of TN, both experimental and clinical studies have begun to implicate immune and inflammatory mechanisms. Altered expression of inflammatory mediators has been reported in trigeminal ganglia, brainstem nuclei, and peripheral nerve tissues in animal models of trigeminal neuropathic pain (Hossain et al., 2017; Bista and Imlach, 2019; Chen et al., 2021). In human studies, changes in circulating cytokine levels have also been described in TN patients; however, these findings have been inconsistent and are limited by the fact that peripheral blood markers may not accurately reflect central pathological processes (Backonja et al., 2008; Liu et al., 2019).

Cerebrospinal fluid (CSF) provides a unique window into the biochemical and immunological environment of the central nervous system and has emerged as a valuable tool for investigating neuroinflammatory mechanisms in neurological and headache disorders (Backonja et al., 2008; Bjurstrom et al., 2016; Lepennetier et al., 2019). CSF immune profiling has revealed disease-specific inflammatory signatures in conditions such as migraine, multiple sclerosis, and small fiber neuropathy, offering mechanistic insights that are not captured by serum-based analyses (Bø et al., 2009). Despite its potential relevance, comprehensive characterization of CSF immune profiles in TN remains scarce (Ostertag et al., 2023).

Therefore, the aim of the present study was to systematically characterize cytokines, chemokines, and growth factors in the CSF of patients with trigeminal neuralgia and to compare these profiles with those of non-pain controls. By focusing on CSF rather than peripheral blood alone, we sought to determine whether TN is associated with a distinct central immune signature, thereby providing new evidence for the involvement of neuroimmune mechanisms in TN pathophysiology.

## 2 Materials and methods

### 2.1 Ethics approval and consent to participate

This study was approved by the Institutional Review Board of the First Affiliated Hospital of Soochow University (IRB No. FAHSU-2023-095). Written informed consent was obtained from all participants for the use of their clinical data and biospecimens in research, in accordance with the Declaration of Helsinki.

### 2.2 Study population

A total of 81 patients with primary trigeminal neuralgia (TN) and 84 control participants (Ctrl) without TN were enrolled in this study between January 2024 and December 2024 at the First Affiliated Hospital of Soochow University. All TN patients fulfilled the diagnostic criteria for primary TN according to the International Classification of Headache Disorders, 3rd edition (ICHD-3), and had complete clinical records. Patients with secondary TN were excluded. Control participants included patients without chronic pain or neurological disease scheduled for removal of lower extremity internal fixation devices, and age-matched healthy individuals undergoing routine health examinations. Patients with systemic inflammatory or autoimmune diseases, active infections, malignancies, or current use of immunomodulatory medications were excluded. Among them, 72 TN patients and 80 controls provided written informed consent. Of these, 21 TN patients and 13 surgical control participants consented to CSF collection, and 59 TN patients and 65 controls consented to serum collection for biomarker analysis.

**FIGURE 1:**
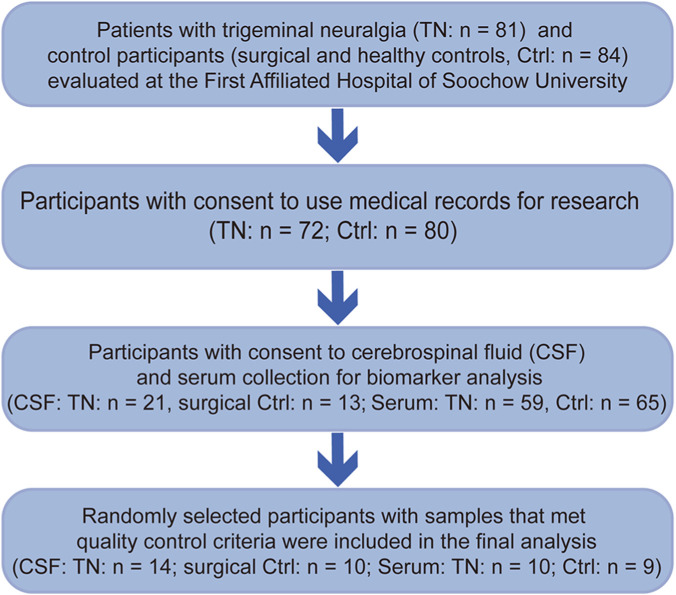
Flowchart of patient selection, biospecimen collection, and final inclusion.

Specimens meeting predefined QC criteria were included in the eligible sample pool (CSF volume ≥3 mL and no visible blood contamination; serum with sufficient volume and no hemolysis). Notably, CSF samples were available exclusively from TN patients and surgical controls. Due to the high per-sample cost of the multiplex assay, biomarker profiling was restricted to a randomly selected subset of this QC-eligible pool. Using computer-generated random identifiers, 14 TN and 10 surgical control CSF samples were selected for analysis. For the serum cohort, a similar random selection was performed; however, one control sample was excluded following the assay due to a markedly deviating cytokine expression profile (outlier), resulting in a final analytical sample size of 10 TN and 9 control serum samples (Figure 1).

### 2.3 Sample collection and processing

CSF collection protocols were tailored to the specific clinical contexts of each group. In the control group, CSF samples were obtained during clinically indicated lumbar punctures performed for spinal anesthesia. In the TN group, CSF was collected during percutaneous balloon compression (PBC) under general anesthesia. For the TN group, samples were obtained only in cases of incidental arachnoid membrane puncture, thereby avoiding additional invasive procedures solely for research purposes.

For both groups, 3 mL of CSF was collected immediately upon subarachnoid access and aliquoted (0.5 mL/tube). Serum samples were collected at admission via peripheral venipuncture (3 mL), allowed to clot for 30 min, and centrifuged at 1,200 × *g* for 10 min. All samples were processed within 3 h at room temperature, temporarily stored at −25 °C for no more than 24 h, and subsequently transferred to −80 °C for long-term storage.

### 2.4 Cytokine/chemokine analysis

The same panel was applied to measure multiple immune mediators in both CSF and serum, utilizing a Luminex assay kit (Catalog No.: LX-MultiDTX-48; R&D Systems). All related assay services were provided by LabEx Biotech Co., Ltd. (Shanghai, China). All assays were performed in a blinded manner regarding group allocation, and, where feasible, samples were processed within the same batch to reduce inter-assay variability. The interleukin (IL)-1 superfamily included IL-1α, IL-1β, IL-1ra, and IL-18; the IL-2 family included IL-2, IL-4, IL-5, IL-7, IL-9, IL-13, IL-15, IL-16, and IL-2Rα; the IL-6 family comprised IL-6 and leukemia inhibitory factor (LIF); the IL-10 family included IL-10; the IL-12 family included IL-12p40 and IL-12p70; and the IL-17 family included IL-17. Interferons were represented by type I IFN-α2 and type II IFN-γ, while the tumor necrosis factor (TNF) superfamily included TNF-α, TNF-β, and TNF-related apoptosis-inducing ligand (TRAIL). CC chemokines included monocyte chemoattractant protein-1 (MCP-1), monocyte chemoattractant protein-3 (MCP-3), macrophage inflammatory protein-1α (MIP-1α), macrophage inflammatory protein-1β (MIP-1β), Regulated upon Activation, Normal T cell Expressed and Secreted (RANTES, CCL5), and eotaxin; whereas CXC chemokines comprised growth-regulated oncogene-α (GRO-α), interleukin-8 (IL-8), monokine induced by interferon-γ (MIG), interferon-γ-inducible protein-10 (IP-10), and stromal cell-derived factor-1α (SDF-1α).

Growth and hematopoietic factors included vascular endothelial growth factor (VEGF), platelet-derived growth factor-BB (PDGF-BB), basic fibroblast growth factor (basic-FGF), β-nerve growth factor (β-NGF), hepatocyte growth factor (HGF), transforming growth factor-β (TGF-β), granulocyte colony-stimulating factor (G-CSF), macrophage colony-stimulating factor (M-CSF), stem cell factor (SCF), granulocyte-macrophage colony-stimulating factor (GM-CSF), and stem cell growth factor-β (SCGF-β).

Other cytokines tested included macrophage migration inhibitory factor (MIF), cutaneous T cell-attracting chemokine (CTACK), and IL-3.

All listed cytokines were detectable in CSF, constituting a comprehensive reference panel for downstream analyses. The same multiplex assay was applied to serum samples. Cytokines with concentrations below the methodological threshold in more than 50% of participants (e.g., IL-5, IL-6, IL-15, and TGF-β) were excluded from serum analyses. For all analyses, cytokine concentrations below the lower limit of detection (LOD) were treated as zero prior to statistical processing.

### 2.5 Statistical analysis

The primary outcome was the difference in cytokine concentrations between TN and Ctrl groups in CSF and serum. Secondary outcomes included associations between cytokines and TN status in logistic regression models, construction of a composite inflammation index, and cytokine network correlations.

Continuous variables are presented as medians (min–max) and compared using two-sided Mann–Whitney U tests; categorical variables were compared using χ^2^ or Fisher’s exact tests, as appropriate.

For cytokine comparisons, false discovery rate (FDR) was controlled using the Benjamini–Hochberg procedure; adjusted p-values are reported as FDR (q-values). A p < 0.05 or q < 0.05 was considered statistically significant.

Logistic regression (family = binomial) was used to estimate associations of individual cytokines with TN (Model 1: unadjusted; Model 2: adjusted for age, sex, and body mass index (BMI)), reporting odds ratios (ORs) with 95% confidence intervals (CIs). For the individual logistic regression analyses, FDR correction was additionally applied to the p values separately for Model 1 and Model 2. To mitigate multicollinearity and overfitting, LASSO (glmnet, R 4.5.0; 10-fold cross-validation; predictors standardized) was applied. The optimal penalty parameter (λ) was selected using λ.min for CSF and λ.1se for serum. Models used a fixed random seed to ensure reproducibility.

For CSF, cytokines with non-zero coefficients at λ.min were z-standardized and weighted by their LASSO coefficients to derive a composite inflammation index; group differences were tested with Mann–Whitney U.

Spearman rank correlation matrices were computed separately for the TN and Ctrl groups in CSF and serum. Pairwise associations were assessed using two-sided Spearman correlation tests and visualized as heatmaps, with the color scale indicating the magnitude and direction of the correlation coefficient. Between-group differences in correlation structure were further assessed using permutation-based differential network testing.

Analyses used Stata 18.0, R 4.5.0, and GraphPad Prism 9.5.0. All tests were two-sided.

## 3 Results

### 3.1 Demographic and clinical characteristics

A total of 33 participants were included in the study. Among them, 14 patients with trigeminal neuralgia (TN) provided cerebrospinal fluid (CSF) samples, of whom 10 also provided paired serum samples. Nineteen non-pain controls were enrolled, including 10 with CSF samples and 9 with serum samples. In the CSF cohort, the median age was 52.5 years in controls and 68.5 years in TN patients. There were no significant differences between groups in sex distribution or BMI (both p > 0.05). TN patients reported severe pain, with a median numerical rating scale (NRS) score of 8, whereas all controls reported an NRS score of 0. In the serum cohort, no significant differences were observed between TN patients and controls with respect to age, sex, or BMI (all p > 0.05). Pain severity, as assessed by NRS scores, showed a distribution consistent with that observed in the CSF cohort. Additional clinical characteristics specific to the TN cohort, including disease duration, pharmacotherapy, prior surgical interventions, TN subtype, and involved nerve divisions, are summarized in Table 1.

**TABLE 1:** Comparison of demographic and clinical characteristics between groups.

Characteristic	CSF		Serum		p value (CSF)	p value (serum)
	Ctrl (n = 10)	TN (n = 14)	Ctrl (n = 9)	TN (n = 10)		
Age^a^	52.5 [35–71]	68.5 [35–82]	62.0 [39–77]	65.5 [35–82]	0.074	0.595
Female, n (%)	5/10 [50.0]	6/14 [42.9]	3/9 [33.3]	5/10 [50.0]	0.120	0.540
BMI	22.29 [16.41–25.97]	22.58 [19.15–29.09]	24.22 [17.79–28.04]	22.38 [19.15–26.45]	0.815	0.414
NRS	0	8 [5–10]	0	8.5 [5–10]	—	—
Disease duration, y	—	4.5 [1–13]	—	3.5 [1–13]	—	—
Classical TN, n (%)	—	14 [100.0]	—	10 [100.0]	—	—
TN medication use^b^, n (%)	—	10 [71.4]	—	8 [80.0]	—	—
Affected division^c^, n	—	0/5/4/5	—	0/4/2/4	—	—
Prior surgery, n (%)	—	7 [50.0]	—	5 [50.0]	—	—

^a^ Data are presented as median [range] for continuous variables and n (%) for categorical variables.

^b^ TN medication use refers to current use of carbamazepine and/or oxcarbazepine at the time of sampling.

^c^ Affected division is presented as V1/V2/V3/multiple.

Abbreviations: CSF, cerebrospinal fluid; TN, trigeminal neuralgia; BMI, body mass index; NRS, numeric rating scale; y, years; —, not applicable. Age and BMI were compared using the Mann–Whitney U test, and sex distribution was compared using the χ^2^ test or Fisher’s exact test when appropriate. A p value <0.05 was considered statistically significant.

### 3.2 Cytokine and chemokine profiles in CSF and serum

**FIGURE 2:**
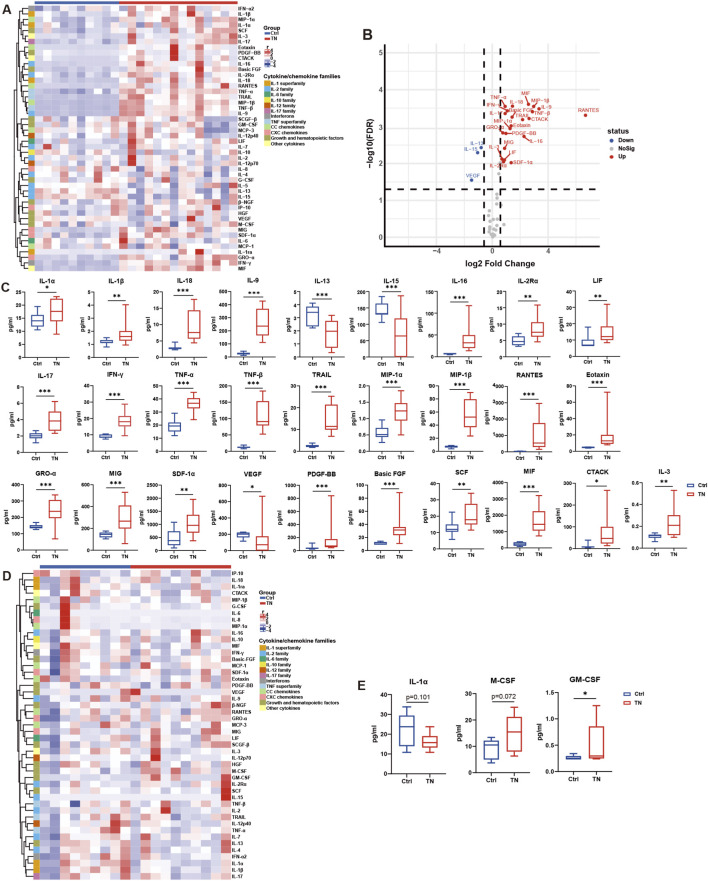
Differential cytokine expression in CSF and serum in control and TN patients. **(A)** Heatmap showing relative cytokine levels in CSF. **(B)** Volcano plot indicating significantly altered cytokines (FDR < 0.05, dashed line indicates fold change = 1.5). **(C)** Box plots of representative CSF cytokines (TN: n = 14, Ctrl: n = 10). **(D)** Heatmap of serum cytokines. **(E)** Box plots of the top three serum cytokines (TN: n = 10, Ctrl: n = 9). Statistical significance was determined using the Mann–Whitney U test (*p < 0.05, **p < 0.01, ***p < 0.001). Abbreviations: Ctrl, control participants; TN, participants with trigeminal neuralgia.

Cytokines showing significant group differences in CSF, along with the three cytokines with the largest between-group differences in serum, are summarized in Figure 2. Complete descriptive statistics for all 47 CSF cytokines and 43 serum cytokines are provided in Supplementary Tables S1, S2.

In CSF, 23 cytokines were significantly elevated in TN patients compared with controls. These included pro-inflammatory cytokines (IL-1β, IL-9, IL-17, IL-18, TNF-α, TNF-β, IFN-γ), regulatory mediators (IL-2Rα, leukemia inhibitory factor [LIF], IL-3), chemokines (MIP-1α, MIP-1β, RANTES, MIG, GRO-α, SDF-1α, eotaxin, CTACK), cytokine-like factors (macrophage migration inhibitory factor [MIF], IL-16), and growth or angiogenic factors (basic FGF, PDGF-BB, stem cell factor [SCF]). In contrast, IL-13, IL-15, and vascular endothelial growth factor (VEGF) were significantly decreased in TN patients (Figures 2B,C).

In serum, granulocyte–macrophage colony-stimulating factor (GM-CSF), macrophage colony-stimulating factor (M-CSF), and IL-1α showed the largest between-group differences; however, only GM-CSF reached statistical significance (p = 0.036; Figure 2E).

Consistent with these findings, heatmap analysis demonstrated broadly elevated CSF cytokine levels in TN patients compared with controls (Figure 2A). Volcano plot analysis further indicated that most significantly altered CSF cytokines exhibited fold changes ≥1.5, with RANTES showing the greatest upregulation, whereas IL-1β was the only cytokine below this threshold (Figure 2B). By contrast, the serum heatmap revealed substantial inter-individual variability and no clear separation between TN and control groups (Figure 2D). Box plots illustrate group distributions for significantly altered CSF cytokines and the top three serum cytokines (Figures 2C,E).

### 3.3 Associations between cytokine/chemokine and TN

Logistic regression analyses identified multiple CSF cytokines nominally associated with TN (Supplementary Table S3). In the unadjusted model (Model 1), 19 cytokines showed significant associations with TN, with most exhibiting positive associations, whereas IL-13 and IL-15 were inversely associated (Table 2).

**TABLE 2:** Logistic regression analysis of CSF cytokines associated with TN.

CSF	Model 1		Model 2	
	OR [95% CI]	p value	OR [95% CI]	p value
IL-1β	69.12 [1.25–3834.82]	0.039*	30.86 [0.62–1530.22]	0.085
IL-9	1.05 [1.00–1.10]	0.047*	1.03 [1.00–1.06]	0.029
IL-13	0.15 [0.03–3.03]	0.016*	0.003 [0.00–3.01]	0.099
IL-15	0.96 [0.93–1.00]	0.044*	0.96 [0.92–1.00]	0.030*
IL-16	1.74 [0.98–3.10]	0.060	1.52 [1.01–2.29]	0.044*
IL-2Rα	2.5 [1.13–5.56]	0.024*	2.33 [1.06–5.11]	0.036*
LIF	1.41 [1.00–1.99]	0.048*	1.42 [0.98–2.07]	0.065
IL-17	5874.95 [1.20–2.87e+07]	0.045*	3601.43 [0.46–2.81e+07]	0.073
IFN-γ	2.98 [0.87–10.28]	0.084	1.81 [1.12–2.94]	0.016*
TNF-α	1.60 [1.07–2.38]	0.021*	1.42 [1.03–2.00]	0.035*
TNF-β	1.10 [1.01–1.20]	0.024*	1.06 [1.01–1.12]	0.026*
TRAIL	2.28 [1.14–4.55]	0.019*	1.87 [1.01–3.47]	0.048*
MIP-1α	928.19 [4.73–182101.40]	0.011*	4922767 [0.04–6.35e+14]	0.106
MIP-1β	1.23 [1.01–1.50]	0.041*	1.12 [1.00–1.24]	0.044*
Eotaxin	4.02 [1.20–13.43]	0.024*	2.48 [1.27–4.86]	0.008*
GRO-α	1.04 [1.00–1.07]	0.026*	1.05 [1.01–1.10]	0.021*
MIG	1.02 [1.00–1.05]	0.035*	1.03 [1.00–1.07]	0.054
SDF-1α	1.00 [1.00–1.00]	0.017*	1.004 [1.00–1.01]	0.045*
SCF	1.27 [1.02–1.57]	0.032*	1.29 [0.99–1.67]	0.055
MIF	1.01 [1.00–1.02]	0.052	1.01 [1.00–1.01]	0.029*
CTACK	1.15 [1.01–1.30]	0.029*	1.13 [0.99–1.29]	0.074

Abbreviations: CSF, cerebrospinal fluid; TN, trigeminal neuralgia; OR, odds ratio; CI, confidence interval; BMI, body mass index. Model 1: unadjusted. Model 2: adjusted for age, sex, and BMI. *p < 0.05.

After adjustment for age, sex, and body mass index (Model 2), 12 cytokines remained positively associated with TN. These included IL-16, eotaxin, TNF-related apoptosis-inducing ligand (TRAIL), TNF-β, IL-9, MIP-1β, macrophage migration inhibitory factor (MIF), TNF-α, IFN-γ, GRO-α, IL-2Rα, and SDF-1α. In contrast, IL-15 retained a negative association with TN (odds ratio [OR] = 0.96, 95% confidence interval [CI] 0.92–1.00, p = 0.030).

**FIGURE 3:**
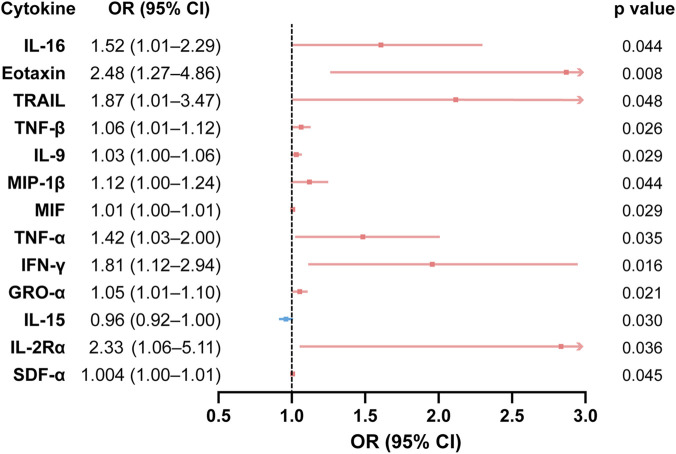
Forest plot of cytokines significantly associated with TN in CSF. Based on multivariable logistic regression (adjusted for age, sex, and BMI). ORs and 95% CIs are shown; p values are two-sided. Abbreviations: CSF, cerebrospinal fluid; TN, trigeminal neuralgia; OR, odds ratio; CI, confidence interval; BMI, body mass index.

Among these cytokines, eotaxin demonstrated the strongest association with TN, corresponding to a 2.48-fold increased odds of TN (95% CI 1.27–4.86). A forest plot depicting cytokines that remained significantly associated with TN in the adjusted model is shown in Figure 3.

However, after FDR correction, none of these nominal associations remained statistically significant; the complete FDR-adjusted results are provided in Supplementary Tables S3.

### 3.4 Composite inflammatory index and its predictive value

**FIGURE 4:**
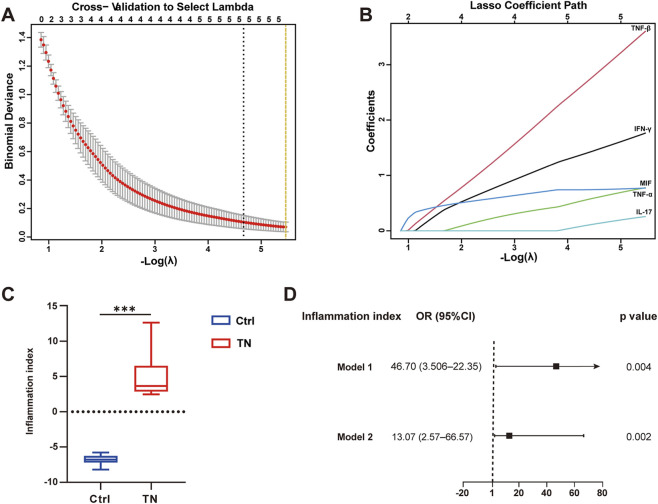
LASSO feature selection and construction of an inflammation index in CSF cytokines. **(A)** Cross-validation curve to select the optimal λ. **(B)** LASSO coefficient paths showing five cytokines retained at λ.min. **(C)** Box plot comparing the inflammation index between TN and control groups (***p < 0.001, two-sided Mann–Whitney U test). **(D)** Forest plot showing logistic regression analysis of the inflammation index for predicting TN. Model 1: unadjusted. Model 2: adjusted for age, sex, and BMI. Abbreviations: CSF, cerebrospinal fluid; TN, trigeminal neuralgia; LASSO, least absolute shrinkage and selection operator; OR, odds ratio; CI, confidence interval; BMI, body mass index.

Following least absolute shrinkage and selection operator (LASSO) feature selection, five cytokines with non-zero coefficients at λ.min were retained, namely, TNF-β, IFN-γ, macrophage migration inhibitory factor (MIF), TNF-α, and IL-17 (Figures 4A,B). All selected cytokines are pro-inflammatory mediators, with TNF-β exhibiting the largest coefficient (Supplementary Table S4).

A composite inflammation index was constructed by standardizing and weighting these cytokines with their LASSO coefficients. The inflammation index was significantly higher in TN patients than in controls (p < 0.001, Figure 4C). Logistic regression analyses further demonstrated that the index remained significantly associated with TN both before and after adjustment for age, sex, and BMI (Model 1: OR = 46.70, 95% CI 3.50–622.35, p = 0.004; Model 2: OR = 13.07, 95% CI 2.57–66.57, p = 0.002; Figure 4D).

In the serum samples, none of the 43 cytokines showed statistically significant associations with TN in either the unadjusted or adjusted logistic regression models; full results are provided in Supplementary Table S5.

**FIGURE 5:**
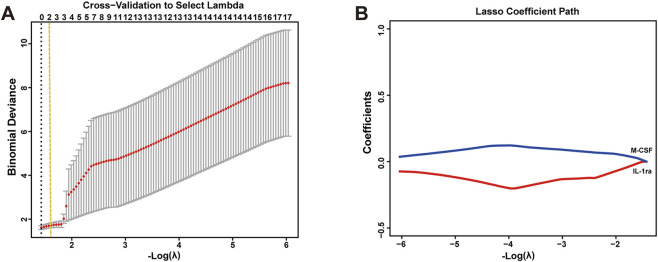
LASSO feature selection in serum cytokines. **(A)** Cross-validation curve to select the optimal λ. **(B)** LASSO coefficient paths showing two cytokines retained at λ.min.

However, LASSO feature selection at λ.min retained only two cytokines: the proinflammatory mediator macrophage colony-stimulating factor (M-CSF) and the anti-inflammatory cytokine IL-1 receptor antagonist (IL-1ra) (Figures 5A,B). Descriptive analyses showed a trend toward higher circulating levels of M-CSF in patients with TN, whereas IL-1ra exhibited the opposite pattern (Supplementary Table S2). Given their opposing biological functions, a composite inflammation index was not constructed. Instead, both cytokines were standardized and simultaneously included in a multivariable logistic regression model adjusted for age, sex, and BMI. Although neither association reached statistical significance, the results suggested a positive association between M-CSF and TN (OR = 2.00, 95% CI 0.92–4.38, p = 0.082) and a negative association between IL-1ra and TN (OR = 0.97, 95% CI 0.93–1.01, p = 0.126) (Table 3).

**TABLE 3:** Logistic regression analysis of serum cytokines retained by LASSO.

Cytokine	OR	95% CI	p value
M-CSF	2.00	[0.92–4.38]	0.082
IL-1ra	0.97	[0.93–1.01]	0.126
Age	0.94	[0.83–1.07]	0.364
Sex	0.33	[0.02–5.71]	0.442
BMI	1.18	[0.59–2.33]	0.641

Abbreviations: CSF, cerebrospinal fluid; TN, trigeminal neuralgia; OR, odds ratio; CI, confidence interval; BMI, body mass index. Cytokines retained by LASSO feature selection were included in the final multivariable models adjusted for age, sex, and BMI.

### 3.5 Altered cytokine correlation patterns in cerebrospinal fluid and serum

**FIGURE 6:**
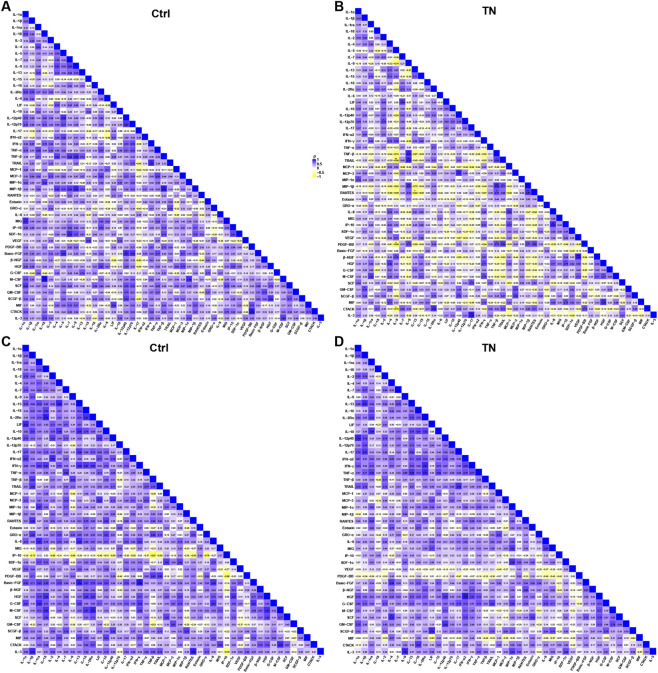
Spearman correlation matrices of cytokines in CSF and serum samples. **(A)** Correlation heatmap of cytokines in CSF from control subjects. **(B)** Correlation heatmap of cytokines in CSF from TN patients. **(C)** Correlation heatmap of cytokines in serum from control subjects. **(D)** Correlation heatmap of cytokines in serum from TN patients. Each square represents the Spearman correlation coefficient (ρ) between cytokine pairs, with blue indicating stronger positive correlations and yellow indicating negative correlations, statistical significance was assessed using two-sided Spearman correlation tests (*p < 0.05, **p < 0.01, ***p < 0.001). Abbreviations: CSF, cerebrospinal fluid; TN, trigeminal neuralgia.

To investigate the overall organization of cytokine interactions, we performed Spearman correlation analysis in both CSF and serum samples. In CSF, control subjects exhibited relatively strong positive correlations among multiple cytokines, suggesting a more coordinated inflammatory network (Figure 6A). In contrast, TN patients displayed generally weaker and more heterogeneous correlations, with a higher proportion of weak or negative associations, consistent with an altered network structure (Figure 6B).

In serum, cytokine correlations were overall stronger than in CSF in both groups (Figures 6C,D). Nevertheless, TN patients also consistently showed attenuated correlation patterns compared with controls. To further assess between-group differences in correlation structure, we performed permutation-based differential network testing, and the corresponding differential correlation heatmaps are shown in Supplementary Figure S1. Overall, these findings suggest that TN may be associated not only with altered inflammatory mediator levels but also with reorganization of cytokine interaction patterns.

## 4 Discussion

In this study, we conducted a comprehensive profiling of inflammatory mediators in cerebrospinal fluid and serum from patients with trigeminal neuralgia. We identified a pronounced upregulation of multiple proinflammatory cytokines in the CSF of TN patients, whereas no individual serum cytokine remained significantly associated with TN after multivariable adjustment. Notably, by integrating cytokines selected through LASSO regression, we constructed a composite CSF inflammatory index that was robustly associated with TN independent of age, sex, and BMI. Collectively, these findings indicate that TN is characterized by a coordinated inflammatory milieu within the central nervous system compartment rather than by a systemic inflammatory response.

Accumulating evidence supports a critical role of neuroinflammation in the pathophysiology of neuropathic pain, including TN (Jiang et al., 2020; Zhang et al., 2024). Proinflammatory cytokines such as TNF-α and IFN-γ are known to enhance neuronal excitability and synaptic transmission by modulating ion channels and neurotransmitter release, thereby facilitating pain transmission (McMahon et al., 2015; Ji et al., 2018; Lu and Gao, 2023). IL-17 has been implicated in glial activation and central sensitization, contributing to persistent pain states (Gao et al., 2009; Kaye et al., 2024). MIF, a pleiotropic cytokine highly expressed in the nervous system, has been shown to sustain inflammatory responses and promote neuropathic pain through both immune and non-immune mechanisms (Alexander et al., 2012; Bavencoffe et al., 2022). TNF-β, produced predominantly by activated lymphocytes, can amplify inflammatory signalling and promote leukocyte recruitment. Prior association studies have also implicated TNF-β genetic variants in migraine-related phenotypes, suggesting that TNF-β–mediated immune activation may be relevant to headache disorders (Trabace et al., 2002; Ishii et al., 2012). Notably, TNF-β contributed prominently to the LASSO-derived composite inflammatory index, highlighting it as a potentially underappreciated component of TN-associated neuroinflammation. Taken together, the concurrent elevation of these mediators in CSF supports the concept that TN involves a complex neuroimmune interaction network within trigeminal pathways and central pain-processing circuits, rather than isolated inflammatory signals. In addition, the reduced CSF VEGF in TN is noteworthy given its established neurotrophic and neuroprotective functions in the central nervous system (Rosenstein et al., 2010; Theis and Theiss, 2018). This finding may reflect impaired trophic or neurovascular support within the trigeminal microenvironment (Lee et al., 2019), although its precise mechanistic significance remains to be elucidated.

A notable finding of our study is the clear compartment-specific pattern of inflammation in TN patients. Whereas robust alterations of cytokine and chemokine profiles were detected in the CSF, serum cytokine and chemokine levels did not show significant associations with TN after adjustment for potential confounders. However, the absence of significant serum findings should be interpreted cautiously, as the limited final serum sample size and substantial interindividual variability may also have reduced statistical power and obscured peripheral associations. This discrepancy suggests that inflammatory processes relevant to TN may be localized to the central nervous system or trigeminal nerve microenvironment. In the periphery, cytokine and chemokine concentrations are influenced by numerous systemic factors, including metabolic status and subclinical inflammation, which may obscure disease-specific signals (Ridker, 2014; Lu et al., 2021; Fang et al., 2022). In contrast, CSF more directly reflects biochemical changes within the central nervous system and has been increasingly recognized as a valuable medium for investigating neuroinflammatory mechanisms in neurological disorders, including chronic headache and facial pain disorders (Ashina et al., 2019; Abu Hamdeh et al., 2020; Abdel-Haleem et al., 2026). Therefore, our findings are more consistent with the hypothesis that neuroinflammation in the central nervous system, rather than systemic inflammation, plays a dominant role in the pathophysiology of TN.

Rather than focusing on individual cytokines or chemokines, we adopted a composite approach to capture the integrated inflammatory network in the CSF. Neuroinflammatory responses are orchestrated by networks of interacting mediators, and a single alteration of cytokine/chemokine may insufficiently reflect such complexity. Using LASSO regression, we identified a subset of proinflammatory cytokines with coordinated elevation in TN patients and constructed a weighted composite inflammatory index. The strong association between this index and TN, independent of demographic factors, suggests that cumulative inflammatory activity may better characterize disease-related neuroimmune activation than any single inflammatory mediator. Nevertheless, the wide confidence intervals observed in regression analyses likely reflect the limited sample size and the exploratory nature of the modelling. Accordingly, the composite index should be interpreted as an integrated inflammatory signature rather than a definitive predictive or diagnostic biomarker.

In serum, LASSO feature selection retained M-CSF and IL-1ra, which exhibited opposing trends in TN patients. M-CSF is involved in monocyte and macrophage activation and has been implicated in chronic pain modulation, whereas IL-1ra acts as an endogenous anti-inflammatory antagonist of IL-1 signalling (Gordon and Plűddemann, 2013; Dinarello, 2018). Given their divergent biological roles, construction of a composite serum index was not biologically appropriate. Although these associations did not reach statistical significance, they may point to subtle systemic immune alterations accompanying TN. Larger studies are required to determine whether peripheral inflammatory mediators contribute to disease susceptibility, or reflect secondary responses to chronic pain, or remain epiphenomena.

Several limitations of this study should be acknowledged. First, the analytical sample size remained limited as only randomly selected subsets of QC-eligible CSF and serum samples were included in the multiplex analyses. This limitation, compounded by substantial inter-individual variability and the large number of analytes tested, may have reduced statistical power, potentially leading to non-significant results in certain comparisons. Therefore, our regression and correlation findings are exploratory and hypothesis-generating. Second, the cross-sectional design precludes causal inference regarding the temporal relationship between inflammation and pain severity. Third, to prioritize patient safety under ethical constraints, CSF collection protocols were not standardized across groups: samples were obtained during spinal anesthesia for surgical controls and during general anesthesia for TN patients. This approach reflected inherent demographic differences between the respective surgical populations and introduced potential confounding from different anesthetic regimens. Although we adjusted for major demographic variables and standardized post-collection processing, residual confounding cannot be entirely excluded. In addition, residual confounding from TN-specific clinical factors, such as disease duration, medication use, prior surgery, and affected division, cannot be excluded. Despite these limitations, our study suggests a coordinated proinflammatory CSF signature in TN and highlights the potential importance of central neuroimmune mechanisms in this disorder. These findings support further investigation of neuroinflammation as a potential therapeutic target and underscore the value of CSF-based biomarkers for advancing the mechanistic understanding of TN.

## Data Availability

The original contributions presented in the study are included in the article/Supplementary Material, further inquiries can be directed to the corresponding authors.

## Glossary

basic-FGF: basic fibroblast growth factor
BMI: body mass index
β-NGF: β-nerve growth factor
CI: confidence interval
CSF: cerebrospinal fluid
CTACK: cutaneous T cell-attracting chemokine
Ctrl: control participants
FDR: false discovery rate
GRO-α: growth-regulated oncogene-α
G-CSF: granulocyte colony-stimulating factor
GM-CSF: granulocyte-macrophage colony-stimulating factor
HGF: hepatocyte growth factor
IL: interleukin
IP-10: interferon-γ-inducible protein-10
IFN-α2: interferon-α2
IFN-γ: interferon-γ
LIF: leukemia inhibitory factor
LOD: lower limit of detection
MCP-1: monocyte chemoattractant protein-1
MCP-3: monocyte chemoattractant protein-3
M-CSF: macrophage colony-stimulating factor
MIG: monokine induced by interferon-γ
MIF: macrophage migration inhibitory factor
MIP-1α: macrophage inflammatory protein-1α
MIP-1β: macrophage inflammatory protein-1β
NRS: numerical rating scale
OR: odds ratio
PBC: percutaneous balloon compression
PDGF-BB: platelet-derived growth factor-BB
RANTES (CCL5): Regulated upon Activation, Normal T cell Expressed and Secreted
SCGF-β: stem cell growth factor-β
SCF: stem cell factor
SDF-1α: stromal cell–derived factor-1α
TGF-β: transforming growth factor-β
TNF-α: tumor necrosis factor-α
TNF-β: tumor necrosis factor-β
TRAIL: TNF-related apoptosis-inducing ligand
VEGF: vascular endothelial growth factor
