# Supplementary material for: Immune profiling in trigeminal neuralgia reveals altered cytokine and chemokine signatures in cerebrospinal fluid and serum
Source: Front Pharmacol. 2026 May 8;17:1796130. doi: 10.3389/fphar.2026.1796130 (PMC13193973; doi:10.3389/fphar.2026.1796130)
Supplement: Supplementary file 1 [file Supplementaryfile1.docx]

**Supplementary Table S1. Cytokine levels in CSF between TN and Ctrl groups.**

| **CSF** | **Ctrl (n = 10)** | | | **TN (n = 14)** | | | **p value** | **FDR** |
| --- | --- | --- | --- | --- | --- | --- | --- | --- |
|  |  | **Median [Min–Max]** |  |  | **Median [Min–Max]** |  |  |  |
| IL-1α | 14.00 [10.55–19.47] | | | 17.62 [8.91–23.27] | | | 0.040 | 0.068 |
| IL-1β | 1.15 [0.80–1.51] | | | 1.60 [0.95–4.03] | | | 0.010 | 0.019 |
| IL-1ra | 96.23 [68.33–111.84] | | | 120.16 [76.02–1747.00] | | | 0.078 | 0.125 |
| IL-18 | 2.78 [2.29–4.65] | | | 7.59 [4.43–17.71] | | | <0.001 | <0.001 |
| IL-2 | 3.06 [2.55–4.29] | | | 2.91 [1.94–8.36] | | | 0.768 | 0.837 |
| IL-4 | 1.04 [0.78–1.78] | | | 0.93 [0.38–1.54] | | | 0.277 | 0.368 |
| IL-5 | 59.49 [55.52–75.98] | | | 49.57 [0.00–144.69] | | | 0.177 | 0.258 |
| IL-7 | 8.06 [3.58–43.38] | | | 12.46 [3.58–41.36] | | | 0.087 | 0.134 |
| IL-9 | 23.14 [11.82–44.43] | | | 236.08 [111.14–427.28] | | | <0.001 | <0.001 |
| IL-13 | 3.42 [2.22–4.12] | | | 1.97 [0.34–3.18] | | | 0.001 | 0.004 |
| IL-15 | 132.55 [105.74–184.80] | | | 64.17 [0.00–187.03] | | | 0.002 | 0.005 |
| IL-16 | 6.61 [4.72–7.90] | | | 32.03 [13.86–117.16] | | | <0.001 | <0.001 |
| IL-2Rα | 4.66 [3.20–7.13] | | | 7.52 [4.57–15.82] | | | 0.003 | 0.008 |
| IL-6 | 2.14 [1.02–4.15] | | | 1.83 [0.00–7.74] | | | 0.639 | 0.714 |
| LIF | 6.61 [6.61–17.93] | | | 12.11 [8.39–31.86] | | | 0.004 | 0.008 |
| IL-10 | 7.81 [5.53–9.37] | | | 6.90 [5.53–11.36] | | | 0.497 | 0.596 |
| IL-12p40 | 47.14 [21.82–74.94] | | | 50.98 [24.17–99.67] | | | 0.513 | 0.601 |
| IL-12p70 | 2.36 [1.53–2.98] | | | 2.48 [1.75–5.67] | | | 0.240 | 0.329 |
| IL-17 | 2.02 [1.17–2.65] | | | 3.84 [2.33–6.22] | | | <0.001 | <0.001 |
| IFN-α2 | 3.71 [1.41–5.44] | | | 4.71 [2.44–8.69] | | | 0.052 | 0.086 |
| IFN-γ | 8.51 [7.39–10.51] | | | 18.00 [9.46–28.75] | | | <0.001 | <0.001 |
| TNF-α | 19.37 [11.96–29.11] | | | 36.72 [24.20–45.08] | | | <0.001 | <0.001 |
| TNF-β | 12.34 [7.77–19.05] | | | 90.87 [52.52–184.21] | | | <0.001 | <0.001 |
| TRAIL | 2.53 [1.76–3.80] | | | 11.42 [6.89–25.23] | | | <0.001 | 0.001 |
| MCP-1 | 147.60 [95.51–211.64] | | | 151.15 [49.86–257.66] | | | 0.977 | 0.977 |
| MCP-3 | 4.32 [3.05–5.56] | | | 3.81 [0.92–7.02] | | | 0.427 | 0.525 |
| MIP-1α | 0.51 [0.27–0.96] | | | 1.24 [0.50–1.85] | | | <0.001 | 0.001 |
| MIP-1β | 6.68 [3.75–8.91] | | | 52.25 [23.40–89.88] | | | <0.001 | <0.001 |
| RANTES | 5.25 [4.31–45.27] | | | 538.07 [150.80–2957.00] | | | <0.001 | <0.001 |
| Eotaxin | 5.00 [3.87–5.33] | | | 12.78 [7.87–72.32] | | | <0.001 | 0.001 |
| GRO-α | 140.46 [125.73–168.41] | | | 237.18 [68.64–337.06] | | | <0.001 | 0.001 |
| IL-8 | 24.02 [14.21–38.85] | | | 20.82 [4.42–28.01] | | | 0.128 | 0.192 |
| MIG | 144.55 [103.51–175.92] | | | 266.90 [60.68–531.14] | | | 0.002 | 0.004 |
| IP-10 | 488.75 [75.15–2021.00] | | | 873.34 [48.31–3188.00] | | | 0.349 | 0.453 |
| SDF-1α | 377.12 [99.18–1081.00] | | | 965.72 [372.01–1955.00] | | | 0.005 | 0.010 |
| VEGF | 201.70 [116.53–226.68] | | | 72.33 [0.00–665.42] | | | 0.016 | 0.028 |
| PDGF-BB | 36.46 [23.93–116.01] | | | 71.96 [44.83–839.33] | | | <0.001 | 0.002 |

**(continued)**

| **CSF** | **Ctrl (n = 10)** | | | **TN (n = 14)** | | | **p value** | **FDR** |
| --- | --- | --- | --- | --- | --- | --- | --- | --- |
|  |  | **Median [Min–Max]** |  |  | **Median [Min–Max]** |  |  |  |
| Basic-FGF | 11.71 [8.91–14.33] | | | 31.38 [10.80–88.52] | | | <0.001 | <0.001 |
| β-NGF | 0.69 [0.46–1.10] | | | 0.89 [0.39–1.21] | | | 0.229 | 0.323 |
| HGF | 278.38 [87.54–620.18] | | | 350.39 [83.53–658.30] | | | 0.38 | 0.479 |
| G-CSF | 33.47 [23.68–66.95] | | | 34.73 [18.89–66.95] | | | 0.769 | 0.821 |
| M-CSF | 12.87 [5.23–20.67] | | | 15.37 [3.11–21.37] | | | 0.558 | 0.638 |
| SCF | 11.99 [5.86–22.49] | | | 17.86 [11.50–34.04] | | | 0.005 | 0.010 |
| GM-CSF | 2.27 [1.19–3.40] | | | 2.56 [0.59–4.56] | | | 0.860 | 0.879 |
| SCGF-β | 25,129.00 [12,177.00–41,729.00] | | | 27,002.50 [15,015.00–45,853.00] | | | 0.815 | 0.850 |
| MIF | 239.09 [82.27–381.03] | | | 1,444.50 [735.42–3204.00] | | | <0.001 | <0.001 |
| CTACK | 7.52 [4.64–39.05] | | | 46.82 [13.99–267.41] | | | <0.001 | <0.001 |
| IL-3 | 0.12 [0.06–0.14] | | | 0.21 [0.10–0.53] | | | 0.004 | 0.009 |

Abbreviations: CSF, cerebrospinal fluid; TN, trigeminal neuralgia; FDR, false discovery rate. Values are presented as medians with ranges (minimum–maximum). Group comparisons were performed using the Mann–Whitney U test, with FDR adjustment for multiple comparisons.

| **Serum** | **Ctrl (n = 9)** | | | **TN (n = 10)** | | | **p value** | **FDR** |
| --- | --- | --- | --- | --- | --- | --- | --- | --- |
|  |  | **Median [Min–Max]** |  |  | **Median [Min–Max]** |  |  |  |
| IL-1α | 23.75 [10.86–33.77] | | | 15.75 [10.86–23.75] | | | 0.101 | 1.000 |
| IL-1β | 3.81 [1.03–6.43] | | | 2.60 [1.56–3.81] | | | 0.111 | 1.000 |
| IL-1ra | 237.44 [87.26–514.62] | | | 159.34 [131.43–372.19] | | | 0.306 | 1.000 |
| IL-18 | 40.66 [5.92–114.91] | | | 40.86 [22.28–85.11] | | | 0.624 | 0.934 |
| IL-2 | 2.86 [1.26–5.41] | | | 2.87 [1.74–11.55] | | | 0.742 | 0.982 |
| IL-4 | 1.30 [0.50–1.84] | | | 1.03 [0.76–2.11] | | | 0.622 | 0.965 |
| IL-7 | 21.32 [6.89–30.30] | | | 22.82 [15.38–42.14] | | | 0.804 | 0.978 |
| IL-9 | 510.45 [466.06–582.40] | | | 514.95 [468.84–564.42] | | | 0.744 | 0.957 |
| IL-13 | 4.44 [0.33–6.78] | | | 1.53 [0.81–9.58] | | | 0.326 | 1.000 |
| IL-16 | 73.82 [11.07–119.46] | | | 78.35 [35.14–188.70] | | | 0.653 | 0.948 |
| IL-2Rα | 45.11 [16.37–84.33] | | | 57.33 [28.24–130.85] | | | 0.253 | 1.000 |
| LIF | 22.77 [6.95–34.57] | | | 24.22 [14.44–43.69] | | | 0.345 | 1.000 |
| IL-10 | 7.36 [1.96–12.75] | | | 8.13 [4.90–17.33] | | | 0.537 | 1.000 |
| IL-12p40 | 41.90 [16.12–97.14] | | | 35.67 [23.99–48.34] | | | 0.360 | 1.000 |
| IL-12p70 | 3.02 [1.45–4.29] | | | 2.54 [2.08–16.58] | | | 0.284 | 1.000 |
| IL-17 | 7.13 [4.54–10.56] | | | 6.84 [5.10–9.29] | | | 0.805 | 0.954 |
| IFN-α2 | 5.96 [1.68–9.47] | | | 4.64 [3.34–7.72] | | | 0.435 | 1.000 |
| IFN-γ | 19.79 [3.32–35.54] | | | 17.58 [7.26–25.52] | | | 0.347 | 1.000 |
| TNF-α | 87.41 [48.14–160.36] | | | 80.83 [56.24–100.55] | | | 0.540 | 1.000 |
| TNF-β | 258.68 [198.50–292.38] | | | 256.56 [238.41–314.25] | | | 0.807 | 0.907 |
| TRAIL | 56.29 [29.27–101.97] | | | 57.12 [28.11–84.03] | | | 0.870 | 0.952 |
| MCP-1 | 49.80 [5.00–80.00] | | | 51.09 [32.75–109.18] | | | 0.539 | 1.000 |
| MCP-3 | 2.14 [0.47–5.66] | | | 1.91 [0.71–5.34] | | | 0.935 | 0.932 |
| MIP-1α | 2.37 [0.71–47.05] | | | 2.13 [1.36–3.53] | | | 0.683 | 0.931 |
| MIP-1β | 184.20 [160.18–261.21] | | | 183.07 [161.60–218.90] | | | 0.775 | 0.969 |
| RANTES | 6890.00 [3993.00–9892.00] | | | 8621.50 [4839.00–9883.00] | | | 0.191 | 1.000 |
| Eotaxin | 51.50 [33.75–161.91] | | | 70.14 [37.52–138.44] | | | 0.514 | 1.000 |
| GRO-α | 336.96 [96.50–650.15] | | | 505.62 [279.04–581.52] | | | 0.288 | 1.000 |
| IL-8 | 8.18 [2.81–183.64] | | | 7.47 [4.49–11.61] | | | 0.683 | 0.931 |
| MIG | 254.75 [112.48–642.57] | | | 334.47 [142.97–872.25] | | | 0.165 | 1.000 |
| IP-10 | 78.99 [2.80–200.58] | | | 74.76 [22.02–196.35] | | | 0.567 | 0.982 |
| SDF-1α | 1098.00 [597.53–1329.00] | | | 1091.00 [855.98–1471.00] | | | 1.000 | 1.000 |
| VEGF | 21.15 [21.15–108.87] | | | 21.15 [21.25–159.41] | | | 1.000 | 1.000 |
| PDGF-BB | 1134.00 [383.88–1904.00] | | | 939.65 [288.45–1773.00] | | | 0.514 | 1.000 |
| Basic-FGF | 57.44 [25.81–90.18] | | | 57.44 [44.22–76.28] | | | 0.680 | 0.957 |
| β-NGF | 0.96 [0.24–1.60] | | | 0.96 [0.43–2.92] | | | 0.594 | 0.991 |
| HGF | 400.24 [272.71–546.78] | | | 410.80 [262.06–557.13] | | | 0.838 | 0.920 |
| G-CSF | 42.66 [12.98–212.47] | | | 36.09 [19.82–61.52] | | | 0.567 | 1.000 |
| M-CSF | 10.50 [3.72–13.38] | | | 15.46 [6.31–24.83] | | | 0.072 | 1.000 |

**Supplementary Table S2. Cytokine levels in serum between TN and Ctrl groups.**

**(continued)**

| **Serum** | **Ctrl (n = 9)** | | | **TN (n = 10)** | | | **p value** | **FDR** |
| --- | --- | --- | --- | --- | --- | --- | --- | --- |
|  |  | **Median [Min–Max]** |  |  | **Median [Min–Max]** |  |  |  |
| SCF | 92.60 [40.77–126.42] | | | 82.52 [47.55–319.02] | | | 0.595 | 0.957 |
| GM-CSF | 0.24 [0.24–0.34] | | | 0.30 [0.24–1.25] | | | 0.036 | 1.000 |
| SCGF-β | 34701.00 [23924.00–43522.00] | | | 40831.50 [31991.00–51665.00] | | | 0.111 | 1.000 |
| MIF | 656.61 [99.36–1597.00] | | | 458.78 [197.70–1069.00] | | | 0.159 | 1.000 |
| CTACK | 661.51 [322.20–1260.00] | | | 607.38 [371.72–982.79] | | | 0.462 | 1.000 |
| IL-3 | 0.33 [0.10–0.52] | | | 0.29 [0.20–0.74] | | | 0.484 | 1.000 |

Abbreviations: TN, trigeminal neuralgia; FDR, false discovery rate. Values are presented as medians with ranges (minimum–maximum). Group comparisons were performed using the Mann–Whitney U test, with FDR adjustment for multiple comparisons.

| **CSF** |  | **Model 1** | | |  |  | **Model 2** | | |  |
| --- | --- | --- | --- | --- | --- | --- | --- | --- | --- | --- |
|  | **OR [95% CI]** | | **p value** | **FDR** | | **OR [95% CI]** | | **p value** | **FDR** | |
| IL-1α | 1.27 [0.99–1.62] | | 0.055 | 0.126 | | 1.30 [0.92–1.83] | | 0.133 | 0.228 | |
| IL-1β | 69.12 [1.25–3834.82] | | 0.039 | 0.144 | | 30.86 [0.62–1530.22] | | 0.085 | 0.170 | |
| IL-1ra | 1.03 [0.99–1.07] | | 0.126 | 0.209 | | 1.03 [0.97–1.09] | | 0.292 | 0.400 | |
| IL-18 | 47.84 [0.09–24966.62] | | 0.226 | 0.350 | | 8.95 [0.91–88.57] | | 0.061 | 0.183 | |
| IL-2 | 1.34 [0.61–2.94] | | 0.463 | 0.585 | | 1.40 [0.64–3.04] | | 0.398 | 0.503 | |
| IL-4 | 0.20 [0.01–3.03] | | 0.246 | 0.358 | | 0.03 [0.00–3.16] | | 0.139 | 0.230 | |
| IL-5 | 0.99 [0.96–1.02] | | 0.480 | 0.576 | | 0.99 [0.96–1.02] | | 0.351 | 0.455 | |
| IL-7 | 1.03 [0.94–1.14] | | 0.480 | 0.591 | | 1.03 [0.93–1.13] | | 0.600 | 0.686 | |
| IL-9 | 1.05 [1.00–1.10] | | 0.047 | 0.133 | | 1.03 [1.00–1.06] | | 0.029 | 0.278 | |
| IL-13 | 0.15 [0.03–3.03] | | 0.016 | 0.384 | | 0.003 [0.00–3.01] | | 0.099 | 0.183 | |
| IL-15 | 0.96 [0.93–1.00] | | 0.044 | 0.141 | | 0.96 [0.92–1.00] | | 0.030 | 0.206 | |
| IL-16 | 1.74 [0.98–3.10] | | 0.060 | 0.131 | | 1.52 [1.01–2.29] | | 0.044 | 0.192 | |
| IL-2Rα | 2.5[1.13–5.56] | | 0.024 | 0.144 | | 2.33 [1.06–5.11] | | 0.036 | 0.192 | |
| IL-6 | 1.04[0.60–1.79] | | 0.910 | 0.929 | | 1.01 [0 57–1.79] | | 0.972 | 0.993 | |
| LIF | 1.41 [1.00–1.99] | | 0.048 | 0.128 | | 1.42 [0.98–2.07] | | 0.065 | 0.173 | |
| IL-10 | 0.91 [0.55–1.51] | | 0.724 | 0.790 | | 0.82 [0.43–1.56] | | 0.551 | 0.678 | |
| IL-12p40 | 1.03 [0.98–1.07] | | 0.279 | 0.394 | | 1.04 [0.98–1.10] | | 0.211 | 0.327 | |
| IL-12p70 | 2.37 [0.58–9.72] | | 0.230 | 0.345 | | 2.36 [0.59–9.46] | | 0.227 | 0.341 | |
| IL-17 | 5874.95[1.20–2.87e+07] | | 0.045 | 0.135 | | 3601.43[0.46–2.81e+07] | | 0.073 | 0.184 | |
| IFN-α2 | 1.96[0.98–3.94] | | 0.058 | 0.121 | | 2.20 [0.90–5.36] | | 0.082 | 0.171 | |
| IFN-γ | 2.98[0.87–10.28] | | 0.084 | 0.149 | | 1.81 [1.12–2.94] | | 0.016 | 0.384 | |
| TNF-α | 1.60 [1.07–2.38] | | 0.021 | 0.202 | | 1.42 [1.03–2.00] | | 0.035 | 0.210 | |
| TNF-β | 1.10 [1.01–-1.20] | | 0.024 | 0.165 | | 1.06 [1.01–1.12] | | 0.026 | 0.312 | |
| TRAIL | 2.28 [1.14–4.55] | | 0.019 | 0.228 | | 1.87 [1.01–3.47] | | 0.048 | 0.177 | |
| MCP-1 | 1.00 [0.98–1.02] | | 0.971 | 0.971 | | 0.99 [0.98–1.01] | | 0.597 | 0.699 | |
| MCP-3 | 0.83 [0.50–1.39] | | 0.484 | 0.567 | | 0.87 [0.47–1.59] | | 0.644 | 0.703 | |
| MIP-1α | 928.19 [4.73–182101.40] | | 0.011 | 0.528 | | 4922767 [0.04–6.35e+14] | | 0.106 | 0.188 | |
| MIP-1β | 1.23[1.01–1.50] | | 0.041 | 0.141 | | 1.12 [1.00–1.24] | | 0.044 | 0.211 | |
| RANTES | 1.02 [1.00–1.05] | | 0.064 | 0.123 | | 1.02 [1.00–1.04] | | 0.094 | 0.180 | |
| Eotaxin | 4.02 [1.20–13.43] | | 0.024 | 0.192 | | 2.48 [1.27–4.86] | | 0.008 | 0.384 | |
| GRO-α | 1.04 [1.00–1.07] | | 0.026 | 0.139 | | 1.05 [1.01–1.10] | | 0.021 | 0.336 | |
| IL-8 | 0.90 [0.79–1.01] | | 0.082 | 0.151 | | 0.87 [0.75–1.01] | | 0.077 | 0.176 | |
| MIG | 1.02[1.00–1.05] | | 0.035 | 0.140 | | 1.03 [1.00–1.07] | | 0.054 | 0.185 | |
| IP-10 | 1.00 [1.00–1.00] | | 0.341 | 0.455 | | 1.00 [1.00–1.00] | | 0.643 | 0.718 | |
| SDF-1α | 1.00 [1.00–1.00] | | 0.017 | 0.272 | | 1.004 [1.00–1.01] | | 0.045 | 0.180 | |
| VEGF | 1.00 [0.99–1.00] | | 0.281 | 0.385 | | 1.00 [0.99–1.00] | | 0.230 | 0.335 | |
| PDGF-BB | 1.06 [1.00–1.12] | | 0.063 | 0.126 | | 1.05 [0.99–1.11] | | 0.078 | 0.170 | |
| Basic-FGF | 1.62 [1.00–2.62] | | 0.051 | 0.129 | | 3.73 [0.34–40.48] | | 0.280 | 0.395 | |
| β-NGF | 11.72 [0.26–524.24] | | 0.204 | 0.326 | | 18.73 [0.21–1705.18] | | 0.203 | 0.325 | |

**Supplementary Table S3. Logistic regression of cytokines in CSF for TN risk.**

**(continued)**

Abbreviations: CSF, cerebrospinal fluid; TN, trigeminal neuralgia; OR, odds ratio; CI, confidence

interval; BMI, body mass index. Model 1: unadjusted. Model 2: adjusted for age, sex, and BMI.

| **CSF** |  | **Model 1** | | |  |  | **Model 2** | | |  |
| --- | --- | --- | --- | --- | --- | --- | --- | --- | --- | --- |
|  | **OR [95% CI]** | | **p value** | **FDR** | | **OR [95% CI]** | | **p value** | **FDR** | |
| HGF | 1.00 [1.00–1.01] | | 0.396 | 0.514 | | 1.00 [0.99–1.01] | | 0.974 | 0.974 | |
| G-CSF | 0.98 [0.92–1.05] | | 0.571 | 0.653 | | 0.98 [0.91–1.05] | | 0.553 | 0.664 | |
| M-CSF | 1.05 [0.89–1.23] | | 0.592 | 0.661 | | 0.99 [0.80–1.24] | | 0.946 | 0.987 | |
| SCF | 1.27 [1.02–1.57] | | 0.032 | 0.140 | | 1.29 [0.99–1.67] | | 0.055 | 0.176 | |
| GM-CSF | 1.13 [0.49–2.63] | | 0.777 | 0.829 | | 1.77 [0.61–5.08] | | 0.296 | 0.395 | |
| SCGF-β | 1.00[1.00–1.00] | | 0.835 | 0.871 | | 1.00 [1.00–1.00] | | 0.881 | 0.940 | |
| MIF | 1.01 [1.00–1.02] | | 0.052 | 0.125 | | 1.01 [1.00–1.01] | | 0.029 | 0.232 | |
| CTACK | 1.15 [1.01–1.30] | | 0.029 | 0.139 | | 1.13 [0.99–1.29] | | 0.074 | 0.178 | |
| IL-3 | 8.15e+17 [0.00–2.67e+39] | | 0.103 | 0.177 | | 1.77e+59 [0.00–4.3e+121] | | 0.063 | 0.178 | |

**Supplementary Table S4. LASSO coefficients of selected cytokines in CSF.**

| **Cytokine** | **Coefficient** |
| --- | --- |
| TNF-β | 3.60 |
| IFN-γ | 1.76 |
| MIF | 0.78 |
| TNF-α | 0.77 |
| IL-17 | 0.27 |

Cytokines with non-zero coefficients retained by LASSO feature selection at the optimal λ (λ.min). Positive coefficients indicate higher cytokine values are associated with higher odds of TN (on the log-odds scale); negative coefficients indicate lower odds. No negative coefficients were retained at the selected λ.

**Supplementary Table S5. Logistic regression of cytokines in serum for TN risk.**

| **Serum** |  | **Model 1** | | |  |  | **Model 2** | | |  |
| --- | --- | --- | --- | --- | --- | --- | --- | --- | --- | --- |
|  | **OR [95% CI]** | | **p value** | **FDR** | | **OR [95% CI]** | | **p value** | **FDR** | |
| IL-1α | 0.86 [0.72–1.02] | | 0.077 | 1.000 | | 0.85 [0.69–1.05] | | 0.125 | 1.000 | |
| IL-1β | 0.53 [0.23–1.23] | | 0.139 | 1.000 | | 0.46 [0.17–1.28] | | 0.137 | 1.000 | |
| IL-1ra | 0.99 [0.98–1.00] | | 0.266 | 0.921 | | 0.99 [0.98–1.01] | | 0.277 | 1.000 | |
| IL-18 | 1.00 [0.97–1.04] | | 0.978 | 1.000 | | 1.00 [0.96–1.04] | | 0.987 | 0.987 | |
| IL-2 | 1.11 [0.71–1.74] | | 0.632 | 0.917 | | 1.23 [0.71–2.14] | | 0.456 | 0.855 | |
| IL-4 | 0.98 [0.12–8.35] | | 0.988 | 0.988 | | 1.09 [0.11–10.95] | | 0.939 | 1.000 | |
| IL-7 | 1.03 [0.92–1.16] | | 0.580 | 0.870 | | 1.06 [0.92–1.23] | | 0.423 | 0.865 | |
| IL-9 | 1.00 [0.98–1.03] | | 0.877 | 1.012 | | 1.00 [0.97–1.03] | | 0.795 | 1.000 | |
| IL-13 | 0.85 [0.59–1.23] | | 0.394 | 0.933 | | 0.86 [0.59–1.26] | | 0.438 | 0.857 | |
| IL-16 | 1.01 [0.98–1.04] | | 0.449 | 0.878 | | 1.02 [0.98–1.05] | | 0.343 | 0.965 | |
| IL-2Rα | 1.03[0.98–1.08] | | 0.227 | 1.000 | | 1.03 [0.97–1.11] | | 0.315 | 1.000 | |
| LIF | 1.06 [0.95–1.18] | | 0.304 | 0.977 | | 1.09 [0.96–1.25] | | 0.178 | 1.000 | |
| IL-10 | 1.15 [0.87–1.53] | | 0.321 | 0.903 | | 1.18 [0.86–1.63] | | 0.302 | 1.000 | |
| IL-12p40 | 0.96 [0.90–1.03] | | 0.263 | 0.986 | | 0.96 [0.89–1.04] | | 0.355 | 0.888 | |
| IL-12p70 | 1.14[0.76–1.71] | | 0.519 | 0.834 | | 1.25 [0.73–2.15] | | 0.422 | 0.904 | |
| IL-17 | 0.91 [0.52–1.58] | | 0.732 | 0.915 | | 0.94 [0.52–1.69] | | 0.835 | 1.000 | |
| IFN-α2 | 0.84 [0.53–1.32] | | 0.442 | 0.947 | | 0.92 [0.55–1.52] | | 0.737 | 1.000 | |
| IFN-γ | 0.95 [0.84–1.08] | | 0.447 | 0.914 | | 0.96 [0.82–1.12] | | 0.600 | 0.900 | |
| TNF-α | 0.98 [0.94–1.02] | | 0.313 | 0.939 | | 0.96 [0.91–1.02] | | 0.211 | 1.000 | |
| TNF-β | 1.01 [0.98–1.05] | | 0.487 | 0.843 | | 1.01 [0.97–1.06] | | 0.532 | 0.921 | |
| TRAIL | 1.00[0.95–1.05] | | 0.917 | 0.983 | | 0.99 [0.94–1.05] | | 0.809 | 1.000 | |
| MCP-1 | 1.02 [0.98–1.07] | | 0.364 | 0.964 | | 1.03 [0.98–1.08] | | 0.287 | 1.000 | |
| MCP-3 | 0.92 [0.49–1.75] | | 0.809 | 0.958 | | 0.96 [0.45–2.06] | | 0.920 | 1.000 | |
| MIP-1α | 0.85 [0.54–1.34] | | 0.495 | 0.825 | | 0.86 [0.52–1.40] | | 0.539 | 0.898 | |
| MIP-1β | 0.99 [0.95–1.03] | | 0.534 | 0.829 | | 1.00 [0.96–1.05] | | 0.915 | 1.000 | |
| RANTES | 1.00 [1.00–1.00] | | 0.246 | 1.000 | | 1.00 [1.00–1.00] | | 0.403 | 0.907 | |
| Eotaxin | 1.00 [0.98–1.02] | | 0.970 | 1.000 | | 1.01 [0.98–1.04] | | 0.569 | 0.914 | |
| GRO-α | 1.00 [1.00–1.01] | | 0.129 | 1.000 | | 1.00 [1.00–1.01] | | 0.323 | 1.000 | |
| IL-8 | 0.94 [0.81–1.10] | | 0.454 | 0.851 | | 0.94 [0.80–1.11] | | 0.471 | 0.848 | |
| MIG | 1.00 [1.00–1.01] | | 0.186 | 1.000 | | 1.00 [1.00–1.01] | | 0.145 | 1.000 | |
| IP-10 | 1.00 [0.98–1.01] | | 0.709 | 0.912 | | 1.00 [0.98–1.02] | | 0.901 | 1.040 | |
| SDF-1α | 1.00 [1.00–1.01] | | 0.673 | 0.918 | | 1.00 [1.00–1.01] | | 0.572 | 0.888 | |
| VEGF | 1.00[0.98–1.03] | | 0.779 | 0.947 | | 1.00 [0.97–1.03] | | 0.832 | 1.000 | |
| PDGF-BB | 1.00 [1.00–1.00] | | 0.466 | 0.839 | | 1.00 [1.00–1.00] | | 0.352 | 0.932 | |
| Basic-FGF | 0.99 [0.93–1.05] | | 0.682 | 0.903 | | 1.00 [0.93–1.07] | | 0.979 | 1.000 | |
| β-NGF | 3.27 [0.49–21.98] | | 0.223 | 1.000 | | 2.84 [0.34–24.06] | | 0.338 | 1.000 | |
| HGF | 1.00 [0.99–1.01] | | 0.887 | 0.998 | | 1.00 [0.99–1.01] | | 0.843 | 0.998 | |
| G-CSF | 0.98 [0.95–1.02] | | 0.371 | 0.928 | | 0.99 [0.95–1.03] | | 0.630 | 0.915 | |

**(continued)**

| **Serum** |  | **Model 1** | | |  |  | **Model 2** | | |  |
| --- | --- | --- | --- | --- | --- | --- | --- | --- | --- | --- |
|  | **OR [95% CI]** | | **p value** | **FDR** | | **OR [95% CI]** | | **p value** | **FDR** | |
| M-CSF | 1.23 [0.99–1.54] | | 0.064 | 1.000 | | 1.40 [0.97–2.00] | | 0.070 | 1.000 | |
| SCF | 1.00 [0.99–1.02] | | 0.658 | 0.925 | | 1.00 [0.98–1.02] | | 0.839 | 1.000 | |
| GM-CSF | 2291.25 [0.01–7.35e+08] | | 0.232 | 1.000 | | 3068.00 [0.01–1.00e+09] | | 0.215 | 1.000 | |
| SCGF-β | 1.00 [1.00–1.00] | | 0.090 | 1.000 | | 1.00 [1.00–1.00] | | 0.073 | 1.000 | |
| MIF | 1.00 [0.99–1.00] | | 0.212 | 1.000 | | 1.00 [0.99–1.00] | | 0.363 | 0.860 | |
| CTACK | 1.00 [0.99–1.00] | | 0.424 | 0.954 | | 1.00 [0.99–1.00] | | 0.341 | 1.000 | |
| IL-3 | 0.65[0.00–426.26] | | 0.896 | 0.983 | | 0.78 [0.00–879.89] | | 0.944 | 0.988 | |

Abbreviations: CSF, cerebrospinal fluid; TN, trigeminal neuralgia; OR, odds ratio; CI, confidence interval; BMI, body mass index. Model 1: unadjusted. Model 2: adjusted for age, sex, and BMI.

**Figure legends**

**Supplementary** **Figure S1.** **Differential Spearman correlation matrices of cytokines in CSF and serum samples.**

**(**A) Differential correlation matrix of cytokines in CSF between TN patients and control subjects. (B) Differential correlation matrix of cytokines in serum between TN patients and control subjects. Each cell represents the difference in pairwise Spearman correlation coefficients between groups (Δρ = ρ_{TN} − ρ_{Ctrl}). Warm colors indicate stronger correlations in the TN group, whereas cool colors indicate stronger correlations in the control group. Asterisks indicate significant between-group differences identified by permutation-based differential network testing (*p < 0.05, **p < 0.01, ***p < 0.001). Abbreviations: CSF, cerebrospinal fluid; TN, trigeminal neuralgia.
